# Predicting Disengagement to Better Support Outcomes in a Web-Based Weight Loss Program Using Machine Learning Models: Cross-Sectional Study

**DOI:** 10.2196/43633

**Published:** 2023-06-26

**Authors:** Aida Brankovic, Gilly A Hendrie, Danielle L Baird, Sankalp Khanna

**Affiliations:** 1 The Australian e-Health Research Centre Health & Biosecurity Commonwealth Scientific Industrial Research Organisation Brisbane Australia; 2 Human Health Program Health & Biosecurity Commonwealth Scientific Industrial Research Organisation Adelaide Australia

**Keywords:** web-based weight loss program, predicting engagement, machine learning–driven intervention, machine learning, artificial intelligence

## Abstract

**Background:**

Engagement is key to interventions that achieve successful behavior change and improvements in health. There is limited literature on the application of predictive machine learning (ML) models to data from commercially available weight loss programs to predict disengagement. Such data could help participants achieve their goals.

**Objective:**

This study aimed to use explainable ML to predict the risk of member disengagement week by week over 12 weeks on a commercially available web-based weight loss program.

**Methods:**

Data were available from 59,686 adults who participated in the weight loss program between October 2014 and September 2019. Data included year of birth, sex, height, weight, motivation to join the program, use statistics (eg, weight entries, entries into the food diary, views of the menu, and program content), program type, and weight loss. Random forest, extreme gradient boosting, and logistic regression with L1 regularization models were developed and validated using a 10-fold cross-validation approach. In addition, temporal validation was performed on a test cohort of 16,947 members who participated in the program between April 2018 and September 2019, and the remaining data were used for model development. Shapley values were used to identify globally relevant features and explain individual predictions.

**Results:**

The average age of the participants was 49.60 (SD 12.54) years, the average starting BMI was 32.43 (SD 6.19), and 81.46% (39,594/48,604) of the participants were female. The class distributions (active and inactive members) changed from 39,369 and 9235 in week 2 to 31,602 and 17,002 in week 12, respectively. With 10-fold-cross-validation, extreme gradient boosting models had the best predictive performance, which ranged from 0.85 (95% CI 0.84-0.85) to 0.93 (95% CI 0.93-0.93) for area under the receiver operating characteristic curve and from 0.57 (95% CI 0.56-0.58) to 0.95 (95% CI 0.95-0.96) for area under the precision-recall curve (across 12 weeks of the program). They also presented a good calibration. Results obtained with temporal validation ranged from 0.51 to 0.95 for area under a precision-recall curve and 0.84 to 0.93 for area under the receiver operating characteristic curve across the 12 weeks. There was a considerable improvement in area under a precision-recall curve of 20% in week 3 of the program. On the basis of the computed Shapley values, the most important features for predicting disengagement in the following week were those related to the total activity on the platform and entering a weight in the previous weeks.

**Conclusions:**

This study showed the potential of applying ML predictive algorithms to help predict and understand participants’ disengagement with a web-based weight loss program. Given the association between engagement and health outcomes, these findings can prove valuable in providing better support to individuals to enhance their engagement and potentially achieve greater weight loss.

## Introduction

### Background

Chronic conditions, such as cardiovascular disease and diabetes, are the leading cause of poor health and death in Australia [1] and in other high-income countries such as the United States, where 6 in 10 adults have a chronic disease [2]. Obesity, poor diet, and inactivity are some of the leading modifiable risk factors for chronic conditions [3]. In Australia, two-thirds of adults are classified as overweight or obese [4], and this number is expected to increase to more than three-quarters of the adult population by 2030 [5]. Approximately 8% of the total burden of disease and injury in Australia is attributable to overweight and obesity, and 5% is attributable to dietary risk, including inadequate fruit, vegetable, and wholegrain consumption [3]. Data from the 2011 to 2012 National Nutrition and Physical Activity Survey found that most Australians do not consume the recommended amounts of healthy food required for health and well-being, with <4% of adults consuming adequate vegetables and less than one-third meeting the recommended intake for grains [6].

There has been an exponential growth in digital behavioral interventions to prevent chronic diseases and promote health [7]. As a result, many systematic reviews have evaluated digital interventions targeting nutrition, physical activity, sedentary behavior, and obesity. Most reviews focus on the effectiveness of interventions in changing behavior, and unfortunately, few interventions have been able to demonstrate substantial and sustained behavior change. A recent systematic review identified a lack of evaluation of other outcomes in digital interventions, such as reach, engagement, and use of technology within interventions [8]. Given that longer-term behavior change is difficult to achieve, including through digital interventions, and low participant use of digital interventions is a recognized barrier to long-term behavior change success, it is surprising that there has not been much focus on the engagement and use of digital interventions.

### Engagement With Digital Health Interventions

Engagement is thought to be critical to interventions achieving successful behavior change and health improvements. Engagement is defined as “the extent to which, and how, individuals participate in an intervention” [9]. Engagement can be considered in a similar way to the exposure or dose of treatment in medical studies. However, in medical studies, participants generally receive the same dose, but in digital health, engagement with the intervention is usually at the participants’ discretion, and it is more difficult to standardize this exposure [10]. In digital health interventions, greater engagement has generally been associated with greater behavior change and better health outcomes [11-13]. However, some research has also suggested that more engagement does not necessarily equate to greater outcomes; rather, effective engagement should be the goal where engagement is at a level sufficient to achieve the intended health outcomes [14]. Despite the growing application and adoption of technology in health interventions, a persistent challenge remains: engagement deterioration or nonuse attrition [10]. Large number of participants initiate action by enrolling in a program, but the majority stop using the technology beyond the first few weeks, leading to substantial user drop out before completion [10], which impacts the behavior change results they are able to achieve. The lack of sustained engagement also impacts the longer-term effectiveness of programs in terms of significant weight loss and improved health outcomes. The reasons for the lack of extended engagement are not well understood, probably because engagement is often seen as a secondary outcome of studies and strategies to enhance engagement are not well evaluated. The lack of focus on digital intervention use and engagement may be representative of a lack of published research in this area but may also be because of methodological differences between studies [8].

### Application of Machine Learning

Applying machine learning (ML) to digital health and weight loss programs could potentially improve the experience for participants by providing more tailored content, in turn increasing their engagement in the intervention and improving the health outcomes achieved by participants. ML has been applied in various ways to address a range of health conditions, such as the detection of diseases such as dementia in individuals [15], diagnostic decision support for clinicians [16], and automated risk assessment and improved service use for mental health [17]. The application of ML in controlled or clinical health settings has been shown to be useful and effective, but its application in real-world digital health interventions is less well understood [18]. A review published in 2019 identified 8 interventions incorporating ML in a real-world research setting; 1 examined self-efficacy for weight loss and 1 personalized nutrition advice based on glycemic response, but the remaining examples were in other areas of health research such as depression, stress management, and smoking cessation [18]. The authors of this review noted that a limitation of the review was the small sample size of studies that applied ML to real-world applications (the average sample size of studies was 71 people [18]).

This presented an opportunity for this study to address several limitations in the current body of literature, including the application of ML to a real-world weight loss intervention that has weight and engagement data collected at regular intervals from a larger sample of participants compared with previously published research. This study used data from a web-based commercial weight loss program to predict a user’s risk of disengagement during a 12-week program. The ability to predict disengagement results before the end of a program could be particularly useful to identify program participants at risk of premature departure and to inform when additional support could be directed toward at-risk individuals, possibly increasing the overall success of the program and outcomes for the participants. Therefore, this study aimed to (1) investigate the ability of state-of-the-art ML algorithms to predict member disengagement week by week, which would allow better support of participants and possibly better weight loss outcomes, and (2) explain the output of the developed predictive models.

## Methods

### Study Design

The participants were adults (aged ≥18 years) who joined the Commonwealth Scientific and Industrial Research Organisation (CSIRO) Total Wellbeing Diet Online program between October 2014 and September 2019. CSIRO Total Wellbeing Diet Online [19] is a web-based 12-week commercial weight loss program managed by Digital Wellness and is available to individuals at a cost of A$199 (US $132) for the first 12 weeks. Some participants opt into additional support from a dietitian in the form of 3 phone or video call consultations (total cost of the 12-week program is A$249 [US $164]). Data were entered by participants into the web-based platform during the registration process (eg, year of birth, sex, postcode, height, and weight at the start of the program) and throughout the program (eg, weekly weight entries). Other data collected within the platform were related to feature use, including entries into the food diary, views of the menu plans, views of exercise plans, views of program content information, forum visits, searches of the food database, and weight entries. These data were provided to the research team in a deidentified format, with each individual member assigned a unique identifier. As part of the registration, participants agreed to their data being used for research purposes; therefore, no direct participant consent was sought.

### Data Description

**Figure 1:**
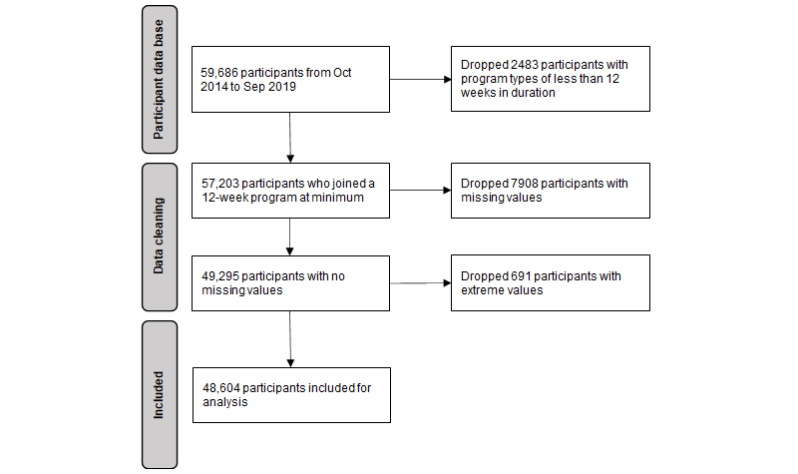
Cohort selection process.

In total, there were 59,686 unique participant IDs over the 5-year study period. This analysis included participants who selected the 12-week weight loss program with or without dietitian assistance. Some participants were members for longer than the initial 12-week program; however, this analysis used data from the first 12 weeks of participation only. After the exclusion of participants with invalid or missing feature entries and those selecting a program type shorter than 12 weeks, the resulting cohort for analysis was 81.43% (48,604/59,686) of all unique participant IDs. Details of the exclusion process are shown in Figure 1.

### Outcome Variables

The primary outcome of this study was the inactivity (disengagement) on the platform. Activity related to the use of 7 features, namely, entries into the food diary, views of the menu plans, views of exercise plans, views of program content information, forum visits, searches of the food database, and weight entries. The outcome of interest was inactivity (no engagement) in the following week, which was defined as the absence of all 7 activity counts within the following week. This resulted in 11 outcome metrics, each corresponding to 1 week of the program, starting from the second week. The absence of any activity on the platform was of interest as it was possibly an indication of imminent withdrawal from the program.

### Predictors

The predictors used in this study were divided into 2 groups: static and dynamic features. The static features included demographic predictors, that is, age; sex; starting BMI; Socioeconomic Indexes for Areas, Index of Relative Socioeconomic Disadvantage decile, with decile 1 representing areas with the most disadvantage or least advantage and decile 10 representing areas with the least disadvantage or most advantage; and predictors anticipated to reflect the motivation for participation, that is, the number of days between setup and nominated start date and the reason (health, family, appearance, or intrinsic or personal) for weight loss. Each reason for weight loss was represented as a categorical variable, with 1 representing its presence and 0 representing its absence. To explore the impact of the opt-in dietitian support, a categorical feature was created and set to 1 for those who chose the program with support from a dietitian and to 0 for those who did not. The dynamic group of features included variables that were subject to change weekly. These included counts of individual activities (diary, forum, menu, exercise plan, food search, program, and weight entry), the sum of individual activities denoted as total activity, and accumulative weight loss. The incremental weight change between 2 adjacent weeks of the program was calculated and included as a predictor. For each predicted outcome metric, starting from week 2, all static and dynamic features available up to that week were considered as predictors. For example, for predicting activity in week 4, all dynamic features available for weeks 1, 2, and 3 were considered alongside the static feature group. The list of all features considered in this study is provided in Table S1 in Multimedia Appendix 1.

### Prediction Models

**Figure 2:**
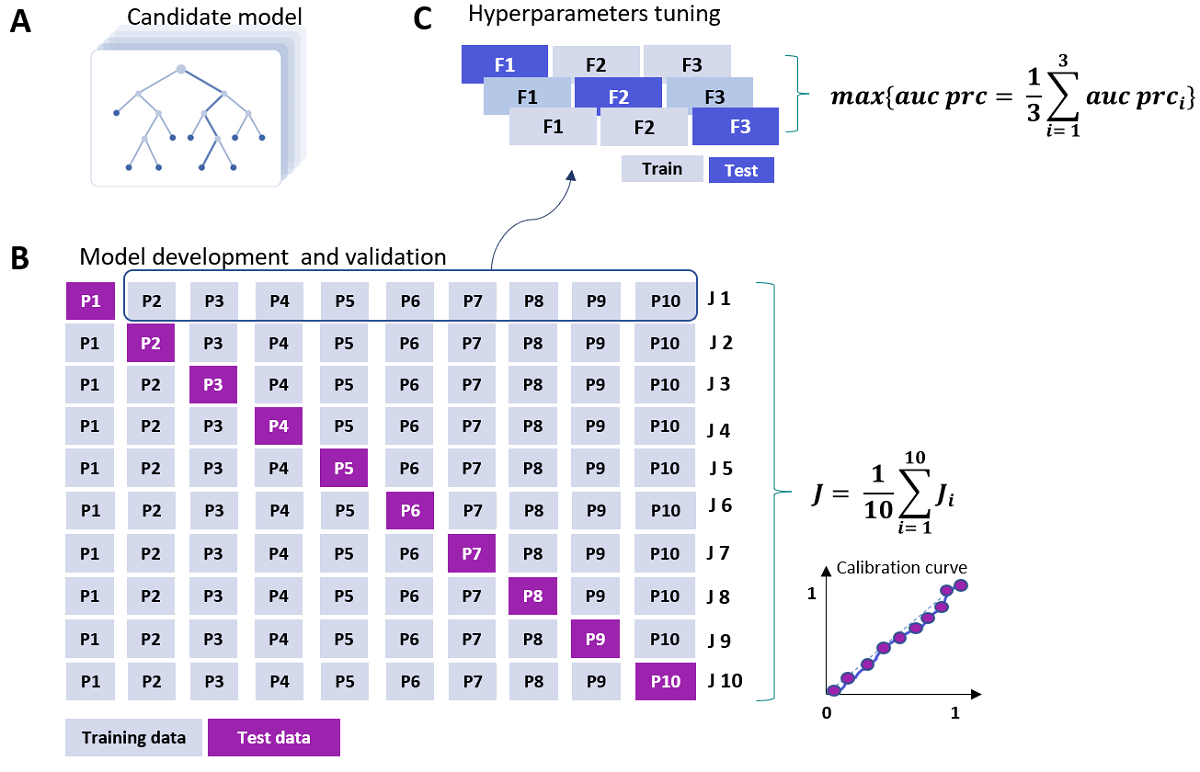
(A) Schematic diagram of tree-based model candidates which takes static and dynamic features and outputs class probability. (B) Schematic diagram of 10-fold cross-validation (10FCV). (C) Schematic diagram of hyperparameters tuning with 3-fold cross-validation on training data belonging to the first fold. P1-S10 indicates data subsets 1 to 10. In magenta (B) and blue (C) are test partitions and, in grey, training partitions. Ji denotes an evaluation metric (in this study area under the precision-recall curve [AUC PRC], area under the receiver operating characteristic curve [AUC-ROC], precision, recall, and F1-score).

In this study, we considered logistic regression with L1 regularization (L1), random forest (RF), and extreme gradient boosting (XGB) as candidate models. L1, popularly known as the least absolute shrinkage and selection operator, was chosen because of its established efficacy in solving prediction problems and ability to perform feature selection to increase model parsimony [20]. The latter ability enhances the predictive accuracy and interpretability of the resulting models and is relevant when considering large predictor sets, where many predictors may not be highly useful for predicting an outcome metric. An advantage of logistic regression is that predictions can be calculated using straightforward mathematics, which makes the implementation of models in a production environment easier than some other approaches. Two tree-based ML approaches, RF and XGB, were used because of their established superiority in pattern recognition from large, complex data [21-23]. The hyperparameters of each of the models were optimized with stratified 3-fold cross-validation on the training partition using an objective function to maximize the area under the precision-recall curve (AUC-PRC; Figure 2). Once found, the best sets of parameters were used to refit the models based on the complete training data. Details of the hyperparameter grids are provided in Table S2 in Multimedia Appendix 1.

### Model Validation

To capture variability, model validation was conducted with nested 10-fold cross-validation (10FCV; Figure 2). Nested cross-validation estimates the generalization error of the underlying model and its hyperparameter search while at the same time preventing the model from yielding an overly optimistic score [24]. Temporal validation was also carried out to simulate reality and check how the model would extrapolate to new data when trained on historic data. For this purpose, data collected between October 2014 and April 2018 were used for training the model, and data from the most recent 18 months (between May 2018 and September 2019) were used for testing.

We did not perform hyperparameter optimization on the training data but instead used the model structure (model hyperparameters) as the best parameters in the 10FCV process.

To evaluate the model performance, we used a set of performance indicators. The area under the receiver operating characteristic curve (AUC-ROC) was used to measure model discrimination. Although AUC-ROC is the gold standard for quantification of the discriminative power of predictive models developed for clinical applications, it is regarded as a misleading performance indicator for imbalanced data sets [15,16]. Therefore, we also computed the AUC-PRC, which has been shown to be more informative than the AUC-ROC [17] in such instances. In addition, we computed recall, precision, and *F*_1-_score metrics, as they are also suitable for assessing model performance where the data are imbalanced. To ensure the reliability of the predictions, calibration curves were considered during the selection process for the final models. Calibration curves were generated by grouping the predictions into quantiles (we used 10), each represented as a point. The x-axis of the calibration curve shows the proportion of true outcomes, and the y-axis shows the mean predicted probability. The better the calibrated model, that is, the more reliable the forecast, the closer the points are along the main diagonal. The equations for computing the evaluation metrics are provided in Multimedia Appendix 1.

### Statistical Analysis

Reported performance means and 95% CIs of 10FCV obtained on test partitions were calculated by averaging and computing the CIs using the Student 2-tailed *t* test. To compare the performance means of the models, one-way ANOVA tests were applied.

### Explainability Module

Unlike regression models, which have an elegant expression that allows direct insight into the model, tree-based ML models require an additional element (the so-called *explainer*) to explain the relationship between the inputs and predicted outputs. To analyze the relevance of predictors in predicting the program participants at risk of disengagement, we created the explainer based on Shapley values introduced in the study by Lundberg et al [25]. Shapley values can be interpreted as the marginal contributions of predictors in predicting class 0 or 1 when compared with the baseline featureless model. Performance of this model was computed as the ratio of samples belonging to a class of interest and total number of samples. Positive values can be interpreted as contributors in predicting class 1, and negative values can be interpreted as contributors in predicting class 0. If computed for only 1 instance, the Shapley values provide a local explanation.

### Software

All models were trained in Python (version 3.6) software. We used the *sklearn* package to implement RF and L1 and the *XGBoost* package to implement the XGB classifier. For hyperparameter tuning, we used the *GridSearchCV* function from Scikit-learn’s *model_selection* module and the *StratifiedKFold* function, with the shuffle set to *true* for model validation. Shapley values used for explanations of the model were calculated with the *SHAP* (Shapley additive explanations) package [26]. The analysis, training, and tests were performed using custom code in Python (version 3.6). Statistical analysis was performed using the Python module *scipy.stats*.

### Ethics Approval

Ethics approval to conduct this research was received from the CSIRO Health and Medical Human Research Ethics Committee (approval 2021_101_LR). A more detailed outline of the weight loss program and data collection process has been published previously [27].

## Results

### Data Characteristics

A demographic summary of the cohort is provided in Table 1. The sample was highly skewed in terms of sex distribution, with 81.5% (39,617/48,604) female, an average age of 49.72 (12.56) years, a starting BMI of 32, and an average Socioeconomic Index for Advantage score of 6.5, meaning that, on average, participants were socioeconomically advantaged.

**Table 1:** Demographic characteristics for the considered cohort as well as the training and test partitions.

Variable name	All members (N=48,604)	Training data (n=31,630)	Test data (n=16,947)
Age (years), mean (SD)	49.72 (12.53)	49.44 (12.56)	50.23 (12.48)
Sex (female), n (%)	39,594 (81.46)	25,950 (82.04)	13,644 (80.51)
Starting BMI (kg/m^2^), mean (SD)	32.43 (6.19)	32.34 (6.23)	32.6 (6.11)
SEIFA^a^ deciles, mean (SD)	6.56 (2.73)	6.61 (2.73)	6.57 (2.73)

^a^SEIFA: Socioeconomic Index for Advantage.

**Figure 3:**
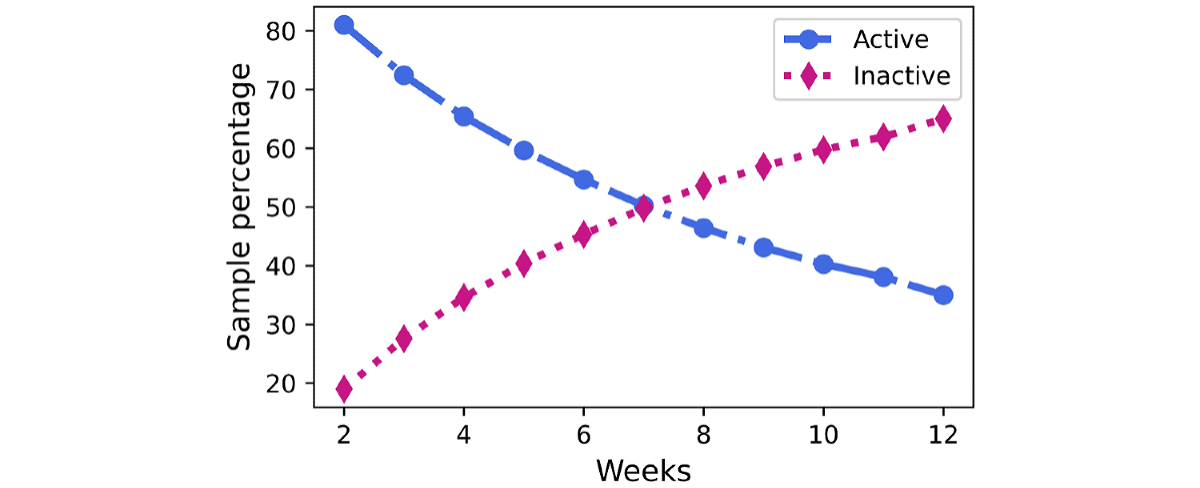
Class distribution over the 12-week program.

The class distribution for each outcome metric in the training and test partitions is shown in Figure 3. In week 2, in total, 19% (9235/48,604) of the participants were inactive. The counts changed exponentially across the weeks, and by week 7, the classes were balanced, meaning that equal number of participants were active and inactive. In week 12, only 35% (17,002/48,604) of the participants were active in the program.

The total number of features used for modeling included 10 static features and 10 dynamic features (feature distributions across the weeks are provided in Figures S1, S2, S3, and S4 in Multimedia Appendix 1) for each outcome metric. Hence, it ranged from 20 for predicting the activity in week 2 to 120 in week 12 of the program.

### Predictive Performance

#### XGB Model Superior

The average area under the curve (AUC), AUC-PRC, precision, recall, *F*_1_-score, and the corresponding 95% CI obtained by the XGB, RF, and L1 models with the 10FCV procedure across the weeks are reported in Table 2. Although XGB has better recall in the later stages of the program compared with the other 2 models, its precision was somewhat deteriorated compared with L1 and RF.

The results showed that predicting activity in week 2 was challenging, and all metrics improved remarkably in week 3. The AUC-ROC performance of all 3 models improved in week 3 from 81% to 88% for L1, 84% to 88% for RF, and 84% to 89% for XGB; for all candidate models, the AUC-PRC performance in week 3 improved by at least 20%. The same performance improvement was observed for the precision, recall, and *F*_1_-score metrics. Overall, all performance metrics improved faster in the first half of the program, after which they continued to improve, albeit at a slower rate.

The computed *P* values are provided in Multimedia Appendix 1. Overall, the results imply that XGB offered the best discrimination. This model was also validated on test data obtained with temporal splitting (Table 3). All the computed metrics were similar to those from 10FCV.

**Table 2:** Performance of XGB^a^, RF^b^, and L1^c^ obtained with 10-fold cross-validation.

			AUC-ROC^d^, mean (95% CI)		AUC-PRC^e^, mean (95% CI)		Precision, mean (95% CI)		Recall, mean (95% CI)		*F*_1_-score, mean (95% CI)
**XGB model**											
	W^f^2	0.85 (0.84-0.85)		0.57 (0.56-0.58)		0.63 (0.62-0.64)		0.41 (0.40-0.42)		0.50(0.48-0.51)	
	W3	0.8 (0.89-0.90)		0.78 (0.78-0.79)		0.77 (0.76-0.78)		0.66 (0.65-0.67)		0.71(0.70 0.72)	
	W4	0.9 (0.90-0.91)		0.85 (0.85-0.85)		0.80 (0.80-0.80)		0.74 (0.74-0.75)		0.77 (0.77-0.78)	
	W5	0.92 (0.92-0.92)		0.89 (0.89-0.89)		0.83 (0.83-0.84)		0.79 (0.79-0.80)		0.81 (0.81-0.82)	
	W6	0.92 (0.92-0.93)		0.91 (0.91-0.91)		0.85 (0.84-0.85)		0.83 (0.82-0.83)		0.84 (0.83-0.84)	
	W7	0.93 (0.93-0.93)		0.93 (0.92-0.93)		0.86 (0.86-0.86)		0.86 (0.85-0.86)		0.86 (0.86-0.86)	
	W8	0.93(0.93-0.94)		0.94 (0.94-0.94)		0.87 (0.87-0.88)		0.88 (0.88-0.88)		0.88 (0.88-0.88)	
	W9	0.94 (0.94-0.94)		0.95 (0.95-0.95)		0.89 (0.88-0.89)		0.89 (0.89-0.90)		0.89 (0.89-0.89)	
	W10	0.94 (0.93-0.94)		0.95 (0.95-0.95)		0.89 (0.88-0.89)		0.90 (0.90-0.91)		0.89 (0.89-0.90)	
	W11	0.93 (0.93- 0.93)		0.95 (0.95-0.95)		0.89 (0.88-0.89)		0.91 (0.91-0.91)		0.90 (0.90-0.90)	
	W12	0.93 (0.93-0.93)		0.95 (0.95-0.96)		0.89 (0.89-0.90)		0.92 (0.91-0.92)		0.91(0.90-0.91)	
**RF model**											
	W2	0.84 (0.83-0.84)		0.55 (0.54-0.57)		0.45 (0.43-0.47)		0.72 (0.71-0.73)		0.55 (0.54-0.57)	
	W3	0.88 (0.88-0.89)		0.77 (0.76-0.78)		0.69 (0.68-0.69)		0.73 (0.72-0.74)		0.71 (0.70-0.71)	
	W4	0.90 (0.90-0.90)		0.84 (0.84-0.85)		0.76 (0.76-0.77)		0.78 (0.77-0.79)		0.77 (0.77-0.77)	
	W5	0.91 (0.91-0.91)		0.88 (0.88-0.89)		0.81 (0.80-0.83)		0.80 (0.78-0.82)		0.81 (0.80-0.81)	
	W6	0.92 (0.92-0.92)		0.91 (0.90-0.91)		0.85 (0.84-0.85)		0.81 (0.79-0.82)		0.83 (0.82-0.83)	
	W7	0.92 (0.92-0.93)		0.92 (0.92-0.93)		0.87 (0.86-0.88)		0.83 (0.82-0.84)		0.85 (0.84-0.85)	
	W8	0.93 (0.93-0.93)		0.94 (0.93-0.94)		0.88 (0.88-0.89)		0.85 (0.84-0.86)		0.87 (0.86-0.87)	
	W9	0.94 (0.93-0.94)		0.95 (0.95-0.95)		0.90 (0.90-0.91)		0.86 (0.85-0.86)		0.88 (0.88 -0.88)	
	W10	0.93 (0.93-0.93)		0.95 (0.95-0.95)		0.91 (0.90-0.91)		0.86 (0.85-0.86)		0.88 (0.88-0.89)	
	W11	0.93 (0.93-0.93)		0.95 (0.95-0.95)		0.91 (0.91-0.91)		0.86 (0.86-0.87)		0.89 (0.88-0.89)	
	W12	0.93 (0.92-0.93)		0.95 (0.95-0.96)		0.92 (0.91-0.92)		0.86 (0.85- 0.87)		0.89 (0.88-0.89)	
**L1 model**											
	W2	0.81 (0.80-0.81)		0.44 (0.43-0.44)		0.34 (0.34-0.35)		0.85 (0.84-0.86)		0.49 (0.49-0.49)	
	W3	0.88 (0.88-0.88)		0.75 (0.74-0.76)		0.75 (0.74-0.75)		0.67 (0.66-0.68)		0.71 (0.70-0.72)	
	W4	0.90 (0.89-0.90)		0.83 (0.82-0.83)		0.79 (0.78-0.79)		0.75 (0.74-0.76)		0.77(0.76-0.77)	
	W5	0.91 (0.90-0.91)		0.87 (0.87-0.88)		0.82 (0.81-0.82)		0.80 (0.79-0.81)		0.81 (0.80-0.81)	
	W6	0.91 (0.91-0.92)		0.89 (0.89-0.90)		0.83 (0.82-0.84)		0.84 (0.83-0.84)		0.83 (0.83-0.84)	
	W7	0.92 (0.92-0.92)		0.91 (0.91-0.92)		0.84 (0.84-0.85)		0.87 (0.87-0.87)		0.86 (0.85-0.86)	
	W8	0.93 (0.92-0.93)		0.93 (0.93-0.93)		0.87 (0.87-0.88)		0.88 (0.87-0.88)		0.87 (0.87-0.88)	
	W9	0.93 (0.93-0.93)		0.94 (0.94 -0.94)		0.89 (0.89-0.89)		0.88 (0.88-0.89)		0.89 (0.88-0.89)	
	W10	0.93 (0.92-0.93)		0.94 (0.94-0.94)		0.89 (0.89-0.90)		0.88 (0.88-0.89)		0.89 (0.89-0.89)	
	W11	0.92 (0.92-0.93)		0.94 (0.94-0.94)		0.90 (0.90-0.90)		0.89 (0.88-0.89)		0.89 (0.89-0.90)	
	W12	0.92 (0.92-0.93)		0.95 (0.94-0.95)		0.91 (0.90-0.91)		0.89 (0.88-0.89)		0.90 (0.89-0.90)	

^a^XGB: extreme gradient boosting.

^b^RF: random forest.

^c^L1: logistic regression with L1 regularization.

^d^AUC-ROC: area under the receiver operating characteristic curve.

^e^AUC-PRC: area under the precision-recall curve.

^f^W: week; the number beside stands for the week of the program and ranges 2 to 12.

**Table 3:** XGB performance evaluation on test data obtained by temporal splitting.

	W^a^2	W3	W4	W5	W6	W7	W8	W9	W10	W11	W12
AUC-ROC^b^	0.84	0.86	0.90	0.91	0.92	0.93	0.93	0.94	0.93	0.93	0.93
AUC-PRC^c^	0.51	0.76	0.83	0.87	0.89	0.92	0.93	0.94	0.94	0.94	0.95
*F*_1_-score	0.48	0.69	0.76	0.79	0.82	0.84	0.86	0.88	0.88	0.89	0.90
Precision	0.59	0.75	0.78	0.80	0.82	0.85	0.86	0.87	0.88	0.88	0.89
Recall	0.41	0.63	0.73	0.78	0.82	0.84	0.87	0.89	0.89	0.90	0.91

^a^W: week; the number beside stands for the week of the program and ranges 2 to 12.

^b^AUC-ROC: area under the receiver operating characteristic curve.

^c^AUC-PRC: area under the precision-recall curve.

#### XGB Calibrates Well at All Probabilities

**Figure 4:**
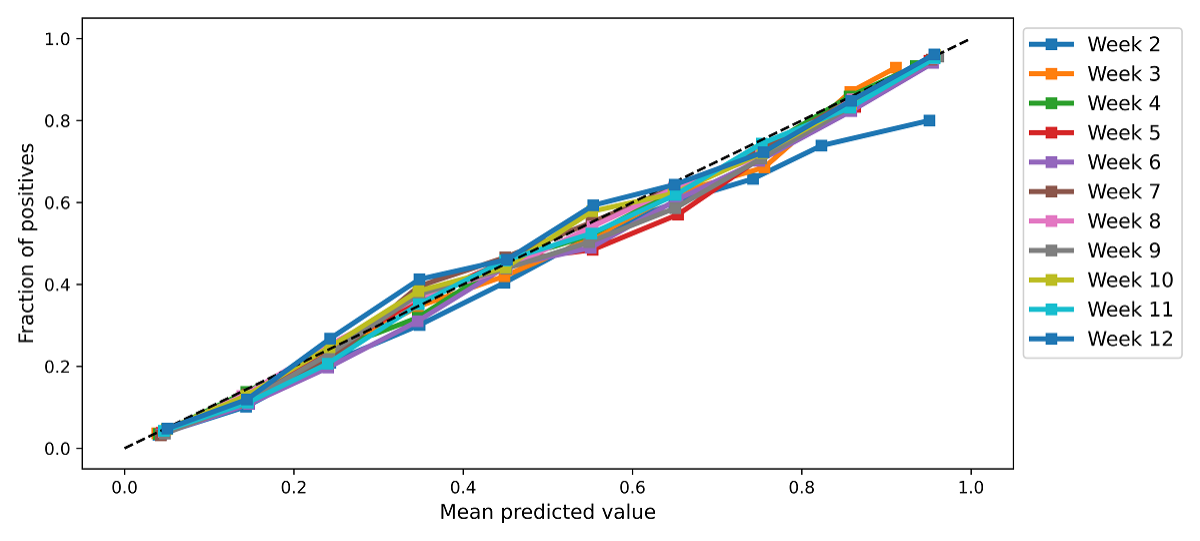
Calibration of the final models on test data from temporal validation.

As a part of the model development process, the calibrations of the final models for L1, RF, and XGB were evaluated. The XGB models had the best calibration across all outcome metrics. Except for the high probabilities in week 2, which were overforecasted, the models calibrated well across the whole probability range for each class and, as such, were considered reliable. Figure 4 shows the calibration plot of the XGB model on the test data obtained with temporal splitting. Better discrimination and good calibration performance led to the recommendation of XGB as the preferred model for future trials in the existing program settings. Receiver operating characteristic curve, precision-recall curve, and calibration curves for all final models obtained on test data acquired through temporal splitting are provided in Figure S5 in Multimedia Appendix 1.

### Explanation of the Model and Relevant Features

**Figure 5:**
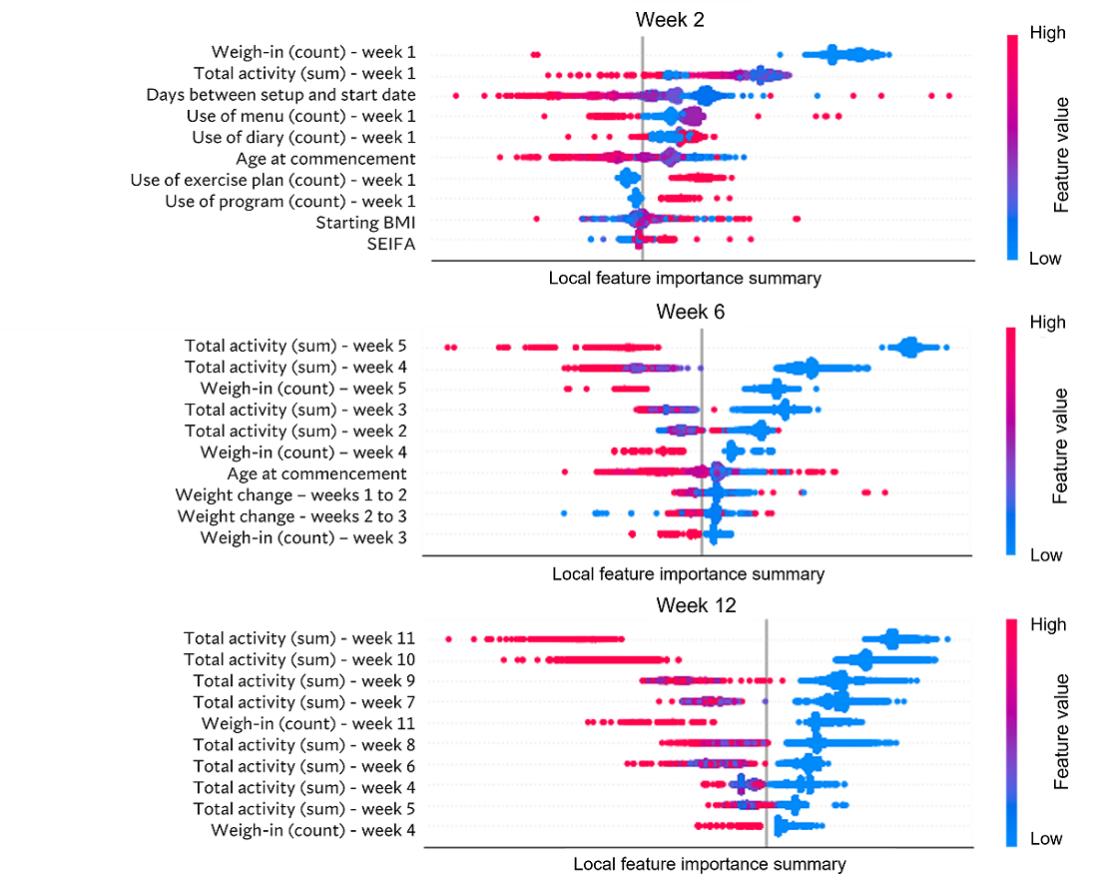
Summary plot of Shapley values computed individually for each program member on test partition for weeks 2, 6, and 12. SEIFA: Socioeconomic Index for Advantage.

To analyze the Shapley value–based feature contribution, we constructed explainers using the training data obtained with the temporal splitting and the XGB models. Summary plots of Shapley values obtained from test data for models predicting activity in weeks 2, 6, and 12 are shown in Figure 5. Features are sorted top-down based on their global contribution. The distance of a dot from the vertical line indicates the feature relevance measured as a contribution to a prediction. Hence, the longer the distance, the more important the feature. A higher concentration of points indicates a larger number of participants with the same or similar Shapley values for that feature. Summary plots obtained for all 11 final models are provided in Figure S6 in Multimedia Appendix 1.

In week 2, the features that contributed the most to predicting the disengagement were entering the week-1 weight, total activity on the platform during the previous week, having the short time between setting up and starting the program, viewing the menu plans, and using the food diary in week 1. As the week progressed, continued activity on the platform and weighing in remained the most important features. Interestingly, in the middle parts of the program (eg, week 6), weight loss (difference between weeks 1 and 2 and weeks 2 and 3) as well as total activity in the previous weeks were important features for predicting disengagement. Further examination of the summary plots showed that higher weight loss pushed the prediction toward engagement (class 0), whereas lower weight loss pushed the prediction toward disengagement (class 1). Participants with fewer days between the application setup and when they were nominated to start the program also pushed the prediction toward engagement.

## Discussion

### Principal Findings

The purpose of this study was to determine how well state-of-the-art ML models predict week-by-week disengagement with a web-based, commercial weight loss program and explain the output of the developed predictive models. To the best of our knowledge, this is the first study to use ML algorithms to predict weekly disengagement in a web-based, real-world weight loss program. Three modeling approaches were tested: the regression-based model L1 and 2 tree-based ML approaches—the RF and XGB models. The models were run to predict disengagement each week during the 12-week weight loss program using all available data from previous weeks. Data about individuals when they signed up at the start of the program were available, as well as individuals’ weekly weight loss and weekly use of 7 features that form part of the web-based platform. Across all predictive models, the results indicated that having a weight recorded in the system and the total activity on the platform from the previous week or weeks were the most important explanatory variables. The performance of the XGB and RF models were similar for the first 3 weeks, although the XGB models performed best overall, with the AUC and AUC-PRC values for the XGB models ranging from 0.85 (95% CI 0.84-0.85) to 0.93 (95% CI 0.93-0.93) and from 0.57 (95% CI 0.56-0.58) to 0.95 (95% CI 0.95-0.96), respectively, across the program. The L1 model systematically had the worst discrimination. The better performance of tree-based methods could be explained by the fact that they inherently include nonlinear effects and interactions, whereas L1 only considers those explicitly included. Good calibration of the final XGB models across all probabilities provides an opportunity to use calibration curves to translate probabilistic output values into susceptibility groups, which can be additionally investigated and used in the future to create additional support for subgroups in greatest need. Depending on the program’s capabilities, this additional support could be delivered via digital media, such as email, SMS text messaging, and app notifications, or with a mix of more traditional media, such as telephone consultation with a health professional. Although the output of the predictive models suggested that having access to additional support from a dietitian via 3 phone or video call consultations was not highly relevant to predicting disengagement, others have suggested that combining human and digital support could help improve engagement [14] or reengage members who have been identified as at risk of further disengagement or dropping out.

### Strengths and Limitations

This study has shown the potential of applying ML algorithms to help predict disengagement with a weight loss program; however, the relationships between the predictors and disengagement should not be considered casual. One key strength of this study was the large sample size. A review of the applications of ML in health reported an average sample size of <100 people [18]; however, this study used data from >48,000 participants of a commercially available weight loss program. We also compared 3 different models that presented good calibration with considerable improvements in predictions over time. One limitation of the models was the challenge of predicting disengagement before week 3 using the activity information collected within the first week of the program along with the considered static features available before starting the program. Furthermore, there were correlations between some of the features, and this study did not investigate the influence of collinearity between some features on the explanations; however, this could be the focus of future research. Finally, the developed models and explainability modules fit well into the existing weight loss program workflow, although they are subject to some adjustment before implementation because of coding changes that may occur in the underlying data.

### Comparison With Prior Work

Many studies have shown a positive association between engagement in digital health programs and better health outcomes for participants [11,12,28,29]. Therefore, the ability to predict when participants will start to disengage is useful for timely intervention to reduce the likelihood of further deterioration in engagement, assuming that participants want to stay engaged and obtain benefits from continuing to engage until program completion. It is recognized that high or early drop out might limit the effectiveness of digital health interventions [30,31], and there have been calls for more research focused on nonuse attrition in digital health programs and to gain a deeper understanding of the deterioration in engagement that occurs before a program is completed and eventual drop out [8,10]. Some reduction in the use of a digital program over time could be expected, as web-based interventions tend to be low intensity and engagement is usually self-directed, so participants might just lose interest if content is not salient and updated regularly [30]. In this study, 50% (24,406/48,604) of the participants did not engage with the platform in week 7 and 65% (31,602/48,604) did not engage in week 12. Similar rates have been reported elsewhere for commercial subscription-based programs [30,32], but nonuse rates as high as 90% after 1 week have been reported on open access websites [33]. Unfortunately, we do not know much about why this is the case and what predicts disengagement or, conversely, longer-term, sustained digital engagement. One study used data mining methods to predict drop out from a commercial digital lifestyle intervention for chronic disease and reported that 2 weeks of platform inactivity (disengagement for 2 weeks) was one of the strongest predictors of drop out [34]. A few other studies have examined disengagement with digital weight loss programs, although not using ML capabilities. Neve et al [13] also used a large sample of data from an Australian commercial weight loss program, albeit a different program, and reported that the behavioral factors that predicted website nonuse were skipping meals and greater levels of emotional eating. Exercising more than once per week and eating breakfast each day protected against website nonuse [13]. Behaviors such as eating breakfast each day and having a regular exercise habit may be indicative of a person who favors structure and routine. Routine behaviors such as regular weighing have also been associated with successful weight loss and weight loss maintenance [35]. Regular self-monitoring through daily or weekly weighing is central to behavioral weight loss programs [35,36], and indeed, having a weight entered into the system in the previous week(s) was also an important variable across all models in the prediction of pending disengagement in this study.

Psychological literature suggests that past behavior is one of the best predictors of future behavior and that the strength of this relationship relates to the period [37]. The results of this study support this notion, whereby total activity on the platform in the previous week(s) was a key variable for the model’s prediction of future disengagement, with the most recent week(s) having greater importance than earlier weeks. A pattern of lower total activity on the platform in the preceding weeks was a strong differentiator of whether someone was disengaged from the platform at a future time point. As few studies focus on engagement in digital health interventions [8], little is known about what predicts engagement or disengagement overall, and even less is known about what predicts engagement throughout the duration of a weight loss program. A study of young adults (aged between 18 and 25 years) combined digital and in-person support within a weight loss program and found that engagement in the first 4 weeks of the program positively predicted overall engagement and weight loss in the program [38]. The authors concluded that monitoring engagement in real time, particularly early on in weight loss programs, may be necessary to effectively intervene [38]. The benefit of the week-by-week ML model presented in this paper is the deeper, temporal understanding of disengagement, which, if applied to a real-world setting, would provide the ability to intervene before the end of the intervention—when in fact, it might be too late. The use of more sophisticated approaches, such as ML techniques, in digital weight loss interventions is an important next step in the evolution of programs to provide greater support to participants when they need it most [39]. The application of these findings means that ML-powered interventions could become more adaptive and facilitate greater weight loss by predicting disengagement earlier and focusing on extending engagement so that participants can realize the intended health benefits.

The use of ML models in real-world health interventions is not common, and very few studies have applied ML in real-world weight loss programs. A review by Triantafyllidis and Tsanas [18] found 8 interventions that incorporated ML in real-life digital health interventions. One of these interventions examined self-efficacy for weight loss, but the others were not weight loss related [18]. The study by Manuvinakurike et al [40] evaluated an automated indexing algorithm to select the most relevant story to provide to participants to have the greatest possible impact on their attitude toward weight loss. Self-efficacy for weight loss increased significantly more in the participants who received content that was tailored using an indexing algorithm compared with random delivery of content [40]. ML approaches have the potential to provide highly tailored content and messages that could maximize engagement and retention without the additional time or effort associated with more traditional, human-driven tailoring approaches. Although not shown in this study, others have suggested that combining digital and human support can facilitate more effective engagement with digital interventions [14]. It might be a case of timing, and further research is required to better understand when, within an intervention, higher engagement is critical; when lower engagement could be equally effective; or when the addition of human support might further facilitate effective engagement [14]. Digital weight loss programs have created new data sources that are well suited to, and could benefit from, the innovation and sophisticated techniques provided by ML approaches [41]. Considering the increasing capacity for more precise tailoring at scale, further research in this area is warranted.

Future research is also required to explore the inclusion of other data such as other lifestyle behaviors and personality characteristics, reported when signing up or during the first few weeks of the program. This could improve the identification of the behavioral patterns of those members prone to disengagement in the early weeks of the program. However, the results imply that the information acquired during week 2 remarkably improved the identification of disengagement. The algorithm used in this study could be improved and generalized to other cohorts with a similar case mix and diet program using similar data. Eventually, the integration of the proposed algorithm into the existing algorithm of the platform would require programing that would activate an appropriate model depending on the week of the program. To overcome this, a dynamic model might be worthy of consideration in future research.

### Conclusions

Given the importance of engagement in achieving behavior change and realizing health improvements, there have been calls for greater emphasis on engagement in digital health research. This study showed the potential of applying ML predictive algorithms to help predict and understand predicted participants’ disengagement with a web-based weight loss program. The most important features for predicting week-by-week disengagement were related to individuals’ total activity on the platform and entering their weight into the platform in the weeks prior. The models developed for this study have evolved in an attempt to fill the gap in understanding the informative relationships between the predictors and disengagement within a web-based weight loss program. This knowledge may help improve retention and better support individuals in engaging in a timely and more effective way. Future research will focus on the evaluation of developed algorithms in a trial, the influence of collinearity between some features on the explanations, and exploiting the potential benefit of including other information available before the program starts, which could possibly improve the prediction of disengagement.

## Appendix

Multimedia Appendix 1 Supplementary information for predicting disengagement to better support outcomes in a web-based weight loss program.

## Abbreviations

10FCV: 10-fold cross-validation
AUC: area under the curve
AUC-PRC: area under the precision-recall curve
AUC-ROC: area under the receiver operating characteristic curve
CSIRO: Commonwealth Scientific and Industrial Research Organisation
L1: logistic regression with L1 regularization
ML: machine learning
RF: random forest
XGB: extreme gradient boosting
