# Supplementary material for: Predicting Disengagement to Better Support Outcomes in a Web-Based Weight Loss Program Using Machine Learning Models: Cross-Sectional Study
Source: J Med Internet Res. 2023 Jun 26;25:e43633. doi: 10.2196/43633 (PMC10337447; doi:10.2196/43633)
Supplement: Multimedia Appendix 1 [file jmir_v25i1e43633_app1.docx]

**Supplementary information for**

**Predicting Disengagement to Better Support Outcomes in an Online Weight Loss Program**

Aida Brankovic^1,*^, Gilly A Hendrie ^2^, Danielle L Baird ^3^, and Sankalp Khanna^1^

^1^CSIRO, The Australian e-Health Research Centre, Brisbane, 4029, Australia

^2^CSIRO, Human Health Program, Adelaide, 5000, Australia

^*^Corresponding author [aida.brankovic@csiro.au](mailto:aida.brankovic@csiro.au)

Contents

[Appendix A List of predictors used in the study 2](#_Toc115100986)

[Appendix B Counts and distribution of the activities over the 12-week program 3](#_Toc115100987)

[Appendix C Hyperparameter tuning 5](#_Toc115100988)

[Appendix D Computation of evaluation metrics 6](#_Toc115100989)

[Appendix E One-way ANOVA test for ROC AUC and AUC PRC means of XGB and RF models 7](#_Toc115100990)

[Appendix F ROC, Calibration and PRC plots of the final models across the weeks 8](#_Toc115100991)

[Appendix G Explainability of the final XGB models across the weeks 9](#_Toc115100992)

# Appendix A List of predictors used in the study

Table S1 lists all predictors available for this study across the weeks.

Table S1 List of predictors

| days_btw_setup_start_dates_trimmed,  Dietitian Flag,  starting_BMI_cleaned,  age_start,  Gender,  weight_loss_health,  weight_loss_appearance,  weight_loss_family,  weight_loss_intrinsic,  SEIFA_deciles,  activity_count.week.1.diary,  activity_count.week.1.forum,  activity_count.week.1.menu,  activity_count.week.1.exercise_plan,  activity_count.week.1.food_search,  activity_count.week.1.program,  activity_count.week.1.weigh_in,  activity_count.week.2.diary,  activity_count.week.2.forum,  activity_count.week.2.menu,  activity_count.week.2.exercise_plan,  activity_count.week.2.food_search,  activity_count.week.2.program,  activity_count.week.2.weigh_in,  activity_count.week.3.diary,  activity_count.week.3.forum,  activity_count.week.3.menu,  activity_count.week.3.exercise_plan,  activity_count.week.3.food_search,  activity_count.week.3.program,  activity_count.week.3.weigh_in,  activity_count.week.4.diary,  activity_count.week.4.forum,  activity_count.week.4.menu,  activity_count.week.4.exercise_plan,  activity_count.week.4.food_search,  activity_count.week.4.program,  activity_count.week.4.weigh_in,  activity_count.week.5.diary,  activity_count.week.5.forum,  activity_count.week.5.menu,  activity_count.week.5.exercise_plan,  activity_count.week.5.food_search,  activity_count.week.5.program,  activity_count.week.5.weigh_in,  activity_count.week.6.diary,  activity_count.week.6.forum,  activity_count.week.6.menu,  activity_count.week.6.exercise_plan,  activity_count.week.6.food_search,  activity_count.week.6.program,  activity_count.week.6.weigh_in,  activity_count.week.7.diary,  activity_count.week.7.forum,  activity_count.week.7.menu, | activity_count.week.7.exercise_plan,  activity_count.week.7.food_search,  activity_count.week.7.program,  activity_count.week.7.weigh_in,  activity_count.week.8.diary,  activity_count.week.8.forum,  activity_count.week.8.menu,  activity_count.week.8.exercise_plan,  activity_count.week.8.food_search,  activity_count.week.8.program,  activity_count.week.8.weigh_in,  activity_count.week.9.diary,  activity_count.week.9.forum,  activity_count.week.9.menu,  activity_count.week.9.exercise_plan,  activity_count.week.9.food_search,  activity_count.week.9.program,  activity_count.week.9.weigh_in,  activity_count.week.10.diary,  activity_count.week.10.forum,  activity_count.week.10.menu,  activity_count.week.10.exercise_plan,  activity_count.week.10.food_search,  activity_count.week.10.program,  activity_count.week.10.weigh_in,  activity_count.week.11.diary,  activity_count.week.11.forum,  activity_count.week.11.menu,  activity_count.week.11.exercise_plan,  activity_count.week.11.food_search,  activity_count.week.11.program,  activity_count.week.11.weigh_in,  TOTAL_ACTIVITY_WEEK1,  TOTAL_ACTIVITY_WEEK2,  TOTAL_ACTIVITY_WEEK3,  TOTAL_ACTIVITY_WEEK4,  TOTAL_ACTIVITY_WEEK5,  TOTAL_ACTIVITY_WEEK6,  TOTAL_ACTIVITY_WEEK7,  TOTAL_ACTIVITY_WEEK8,  TOTAL_ACTIVITY_WEEK9,  TOTAL_ACTIVITY_WEEK10,  TOTAL_ACTIVITY_WEEK11,  running_wt_loss.0,  running_wt_loss.7,  running_wt_loss.14,  running_wt_loss.21,  running_wt_loss.28,  running_wt_loss.35,  running_wt_loss.42,  running_wt_loss.49,  running_wt_loss.56,  running_wt_loss.63,  running_wt_loss.70,  running_wt_loss.77 |
| --- | --- |

#
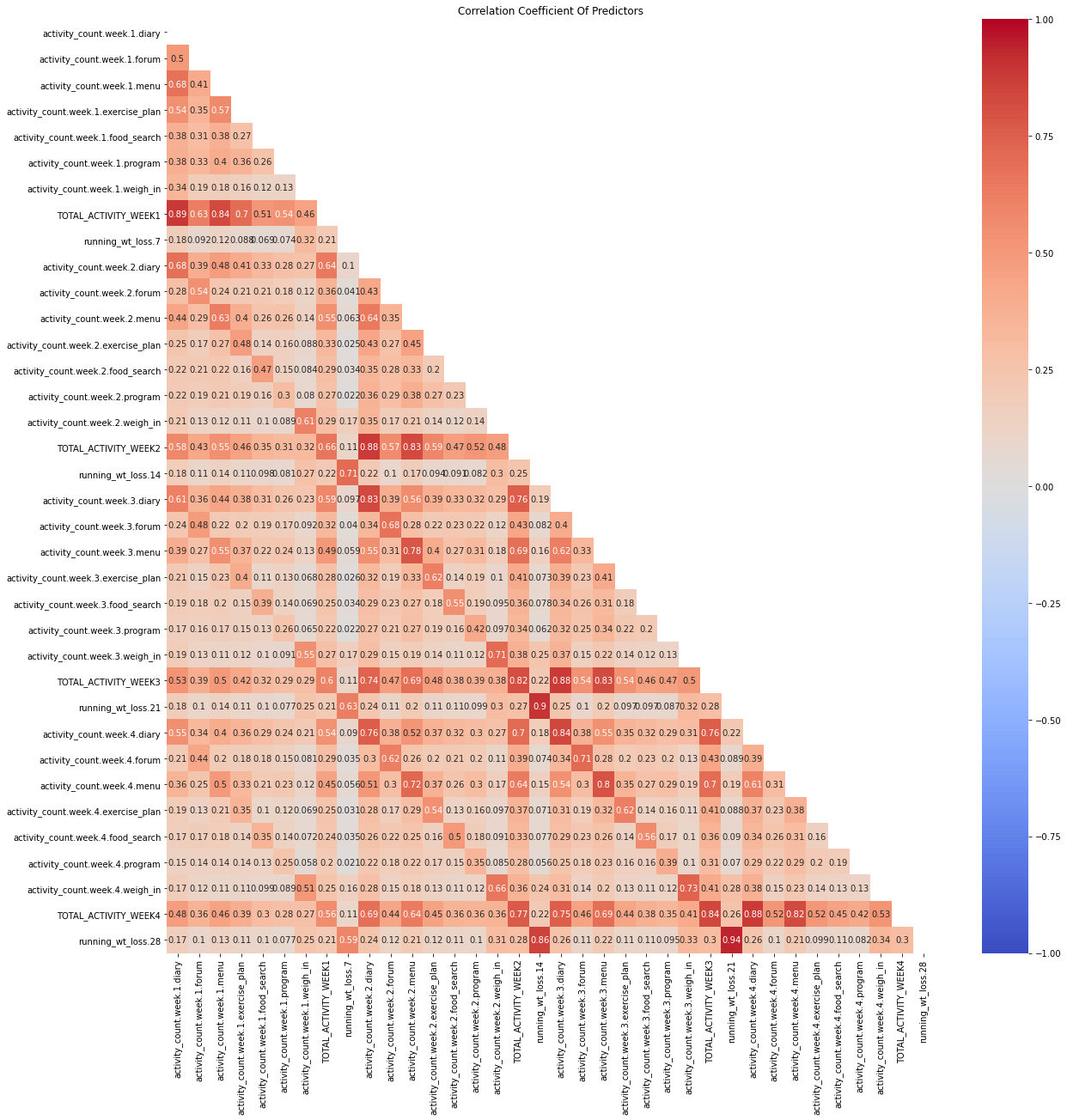


Figure S1 Correlation between the static predictors and predictors of the first 4 weeks of the programme.

Note: Adding correlation plot containing all features would heavily compromise the readability. Though, similar correlation patterns are obtained for the remaining weeks. Correlation plot of dynamics features identified to be highly correlated the weeks is shown in Figure S2.

Moderate to high correlation between Total activity feature and Menu as well as Diary unlike with other activities can be explained as follows: unlike other activities Menu and Dairy are entered more frequently i.e. daily so they contribute the sum the most and hence change in their value cause the change of total activity in the same direction.


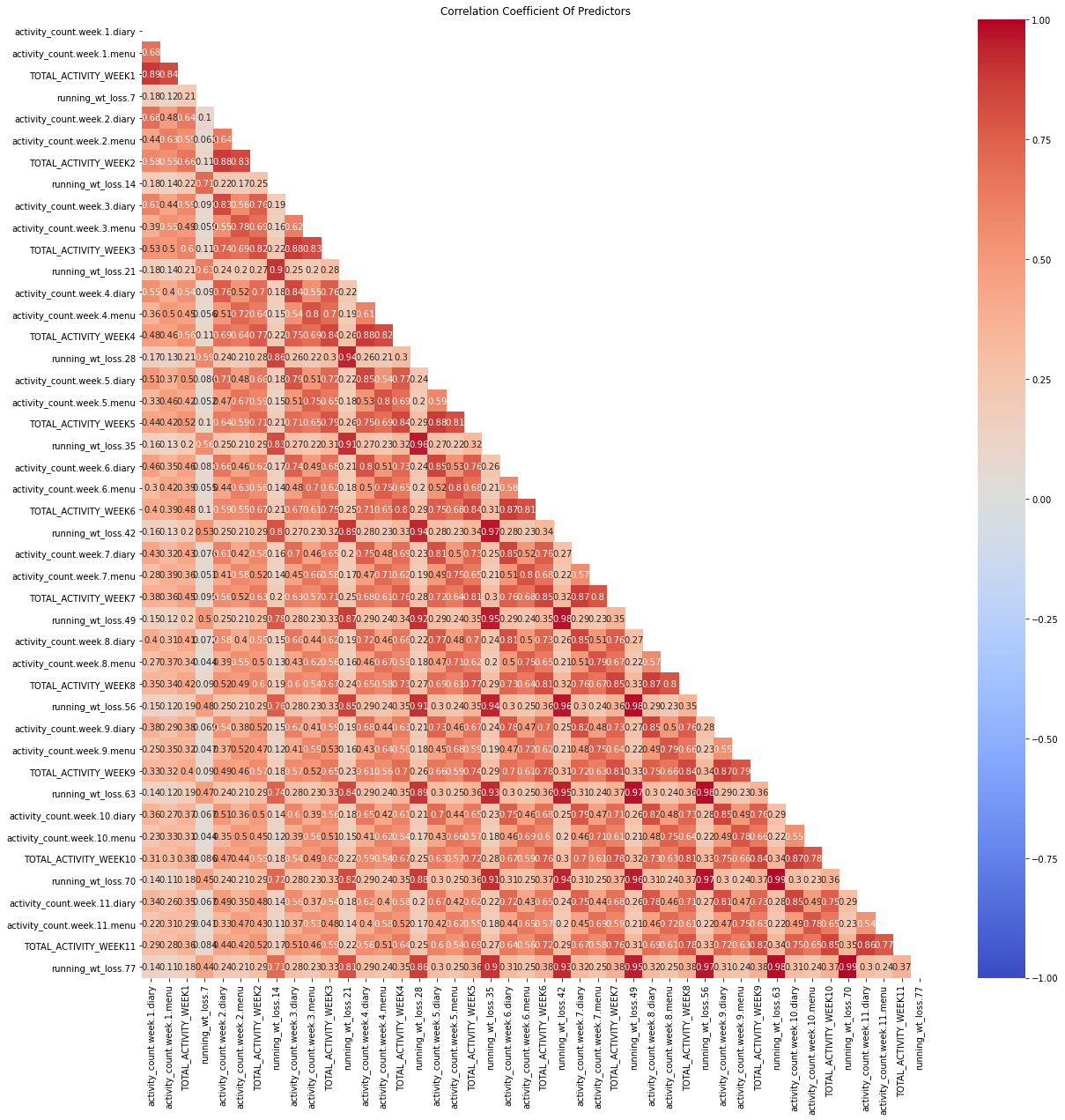


Figure S2 Correlation plot of selected dynamic features across the 12 weeks of the programme.

Note: Only dynamic features identified to have high level of correlation were selected for the plot in Figure S2.

#

# Appendix B Counts and distribution of the activities over the 12-week program


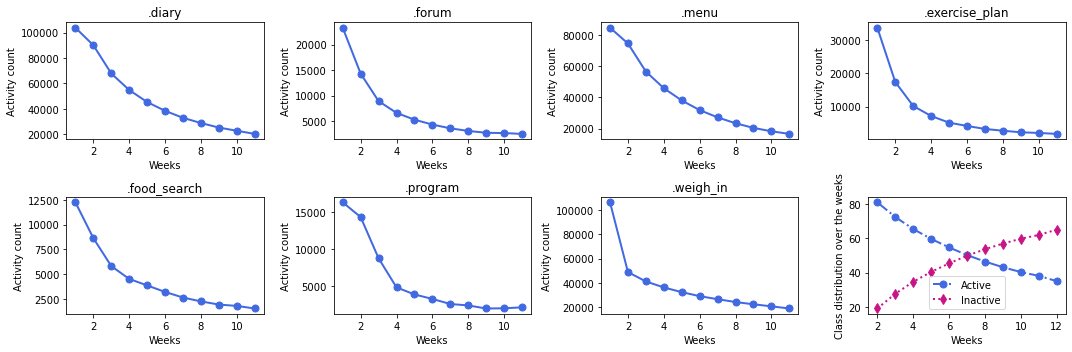


Figure S3 Total Count of each activity over the 12-week program

Figure S4 Diary, forum, menu, exercise plan activities across 12 weeks of the program (top to bottom)


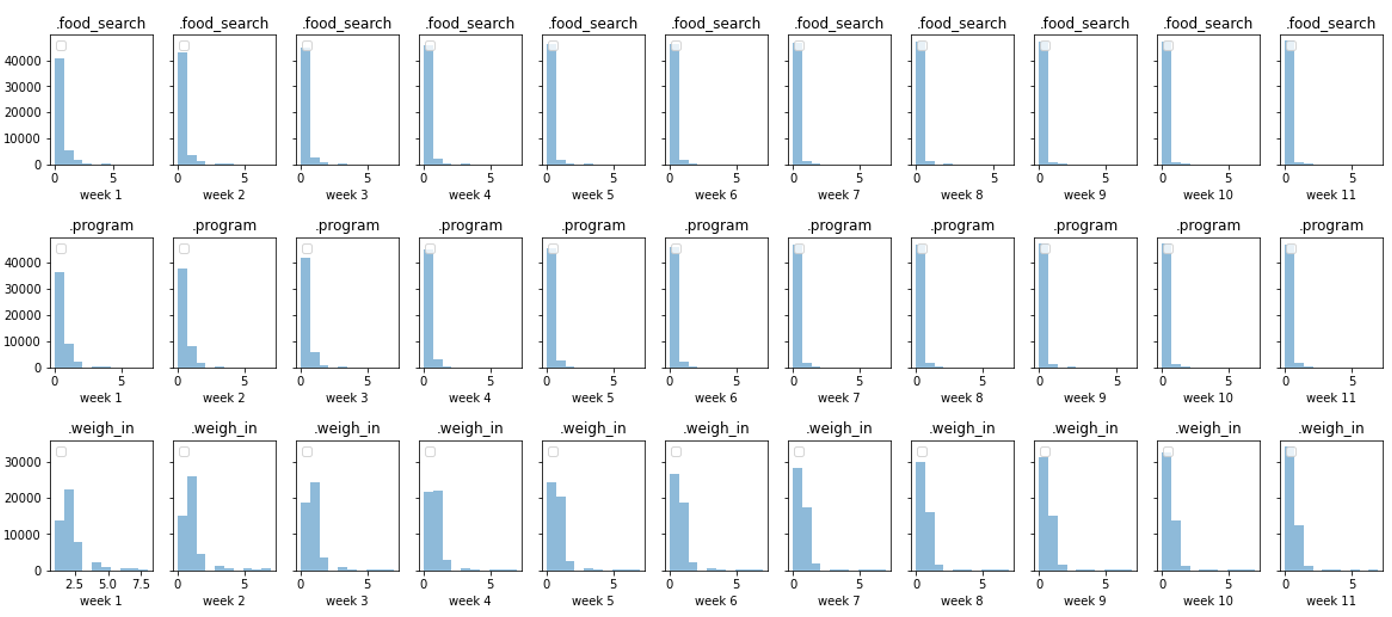


Figure S5 Food search, program and weigh in activities across the weeks of the program


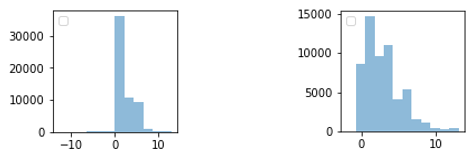


Figure S6 Number of days between set up and nominated start date (left) and number of days between the nominated start date and registration date (right). Cases <-14 and>14 days were removed.

# Appendix C Hyperparameter tuning

Hyperparameter tuning was used to find the best set of hyperparameters for each method to maximise AUC. A grid search over proposed values was used, fitting models to the training data. The grid search was implemented with the sklearn package and GridSearchCV library from the model_selection module. The cross-validation strategy was specified to be stratified 3-fold cross validation with the refit argument set to True which refits the estimator using the parameters found to be best on the whole trainng dataset. Stratified cross validation ensured that all folds contained the same distribution of classes. Values for the grid search were chosen to cover the range 0—1 (for the L1 regularisation) and by domain expertise. Details of the parameter grid for model candidates are presented in Table S2.

Table S2 Hyperparameter grids used for optimization

| MEthod | GRID |
| --- | --- |
| L1 | C = [0.001, 0.005,0.01, 0.03, 0.05, 0.08, 0.1, 0.25, 0.5, 0.8, 1] |
| XGB | max_depth: [3, 5, 7, 9],  n_estimators: [15, 35, 55, 100],  gamma: [0, 10, 50, 1000],  objective: [binary:logistic],  eval_metric: [aucpr] |
| RF | n_estimators = [15, 35, 55, 100]  max_features = [auto]  max_depth = [3, 5, 7]  bootstrap = [True]  class_weight = [balanced, None] |

Parameters of the final model:
XGBClassifier(base_score=0.5, booster=gbtree, colsample_bylevel=1, colsample_bynode=1, colsample_bytree=1, eval_metric=aucpr, gamma=0, gpu_id=-1, importance_type=gain, interaction_constraints=, learning_rate=0.300000012, max_delta_step=0, max_depth=3, min_child_weight=1, missing=nan, monotone_constraints=(), n_estimators=100, n_jobs=8, num_parallel_tree=1, random_state=0, reg_alpha=0, reg_lambda=1, scale_pos_weight=1, subsample=1, tree_method=exact, validate_parameters=1, verbosity=None)

# Appendix D Computation of evaluation metrics

- AUC-ROC is a performance measurement for the classification problems obtained by evaluating the discrimination capabilities of a model given different thresholds. False positive rate is on x-axis and true positive rate on y-axis. Higher the AUC better the model was in correctly predicting a class for a given test sample (e.g., class 0 or 1, in a binary classification).
- Precision is a measure of result relevancy, and it is computed as ratio between the number of true positives ($T_{p}$) over the number of true positives plus the number of false positives ($F_{p}$), i.e.

$Precision= \frac{T_{p}}{T_{p}+F_{p}}$.

- Recall is a measure of how many truly relevant results are returned defined as the number of true positives ($T_{p}$) over the number of true positives plus the number of false negatives ($F_{n}$), i.e.

$Recall= \frac{T_{p}}{T_{p}+F_{n}}$.

- Precision and recall are related to F1-Score, which is defined as the harmonic mean of precision and recall, i.e.

$F1=2 \frac{Precision \times Recall}{Precision+ Recall}$.

- AUC-PRC is just like the ROC-AUC, in that it summarizes the curve with a range of threshold values as a single score. Recall is on x-axis and precision on y-axis. Higher the AUC-PRC better the model in discriminating among the two classes.
- Calibration plot groups predictions into quantiles, typically 10 and represents each as a point such that the proportion of true predictions lies on x-axis and mean predicted probability on y-axis.

# Appendix E One-way ANOVA test for ROC AUC and AUC PRC means of XGB and RF models

| ROC AUC means week 2 F_onewayResult(statistic=0.019805246372790172, pvalue=0.889089931657605)  AUC PRC means week 2 F_onewayResult(statistic=0.007091858677330377, pvalue=0.9334861734970537)  ROC AUC means week 3 F_onewayResult(statistic=0.18344064942288624, pvalue=0.6719602320940017)  AUC PRC means week 3 F_onewayResult(statistic=0.06355890431653101, pvalue=0.8029391624176657)  ROC AUC means week 4 F_onewayResult(statistic=23.007548710956318, pvalue=0.0001444601323358382)  AUC PRC means week 4 F_onewayResult(statistic=4.049989003706569, pvalue=0.059380087372167996)  ROC AUC means week 5 F_onewayResult(statistic=15.94205566344136, pvalue=0.0008534999501317047)  AUC PRC means week 5 F_onewayResult(statistic=6.772217916182202, pvalue=0.018010216379568764)  ROC AUC means week 6 F_onewayResult(statistic=20.323961253729312, pvalue=0.00027202903006963566)  AUC PRC means week 6 F_onewayResult(statistic=3.4741082883517844, pvalue=0.07873191734258156)  ROC AUC means week 7 F_onewayResult(statistic=16.719636205347967, pvalue=0.0006889300921207568)  AUC PRC means week 7 F_onewayResult(statistic=3.1700115272117877, pvalue=0.09188739442767739)  ROC AUC means week 8 F_onewayResult(statistic=3.3002756052154325, pvalue=0.08595874241324382)  AUC PRC means week 8 F_onewayResult(statistic=0.21137722086398486, pvalue=0.6511946725016494)  ROC AUC means week 9 F_onewayResult(statistic=4.03728321311133, pvalue=0.059742660888285996)  AUC PRC means week 9 F_onewayResult(statistic=0.29982651720778963, pvalue=0.5907169407741761)  ROC AUC means week 10 F_onewayResult(statistic=3.187185785736067, pvalue=0.09107884052222576)  AUC PRC means week 10 F_onewayResult(statistic=0.08656625168334238, pvalue=0.7719546913204313)  ROC AUC means week 11 F_onewayResult(statistic=2.8425277414417223, pvalue=0.10905552652342135)  AUC PRC means week 11 F_onewayResult(statistic=0.23344942711278496, pvalue=0.6348024983972513)  ROC AUC means week 12 F_onewayResult(statistic=3.235142024272726, pvalue=0.08886503599100203)  AUC PRC means week 12 F_onewayResult(statistic=0.05114287069125742, pvalue=0.8236344876521283) |
| --- |

# Appendix F ROC, Calibration and PRC plots of the final models across the weeks

| 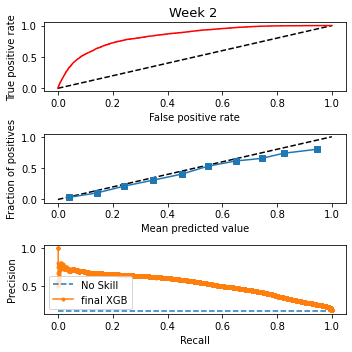 | 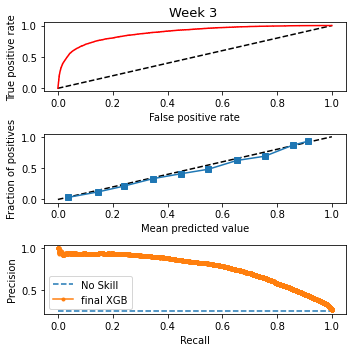 | 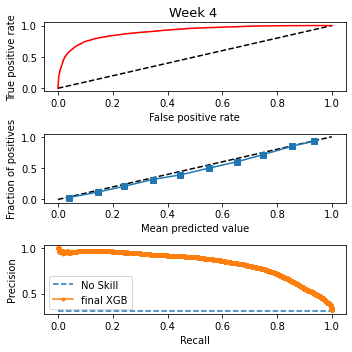 |
| --- | --- | --- |
| 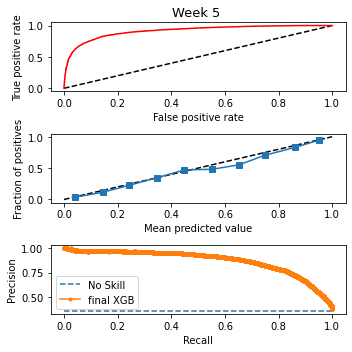 | 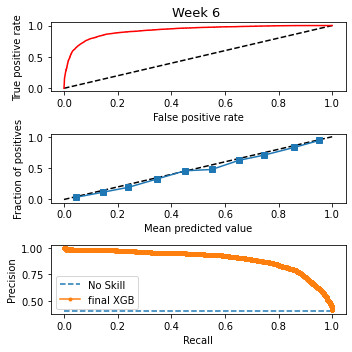 | 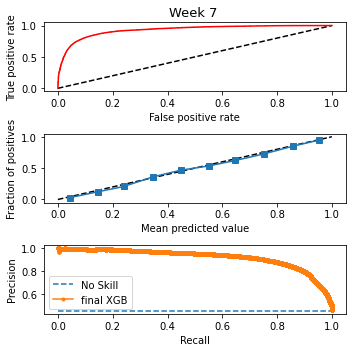 |
| 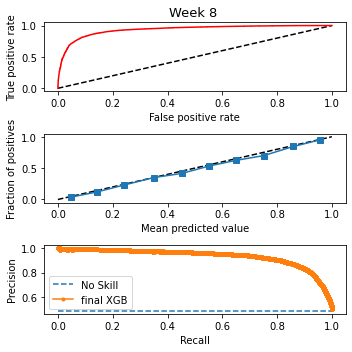 | 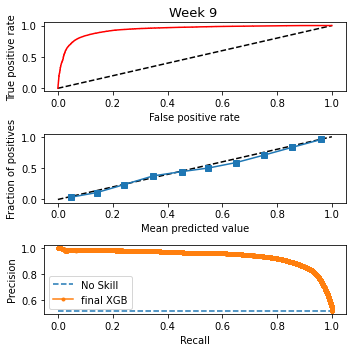 | 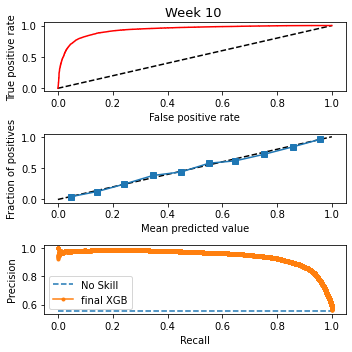 |
| 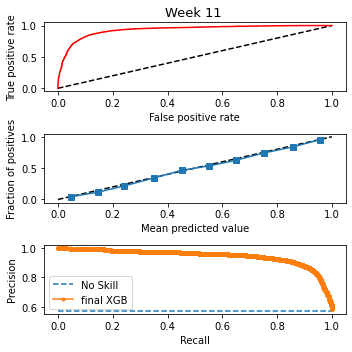 | 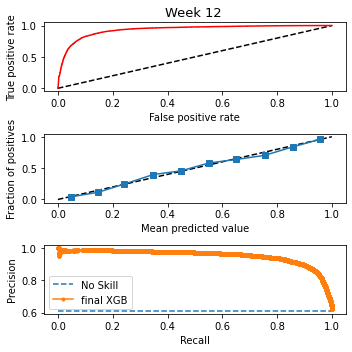 |  |

Figure S7 ROC (red), PRC (orange) and calibration (blue) curves for the final XGB model obtained on the test data obtained in temporal validation procedure (April 2018-Sept 2019).

# Appendix G Explainability of the final XGB models across the weeks

Figures below displays relative feature obtained on test data. Each figure contains 3 pieces of information: 1) Top ten contributors that drive the evolving risk of inactivity in the predicted week ranked top to bottom based on relevance computed on whole test data, 2) Each dot represents one data sample, and its colour indicates the value of the corresponding feature for that data sample. Extremes of the color-bar (magenta and blue) represent the highest and lowest value of the corresponding feature respectively. Thicker dot clouds indicate many samples which have the same or similar Shapley values for that feature. of 3) The distance from the vertical axis is determined by the Shapely value computed for that feature. The more distant point is the grater is Shapely value corresponding to it and thus its importance in the prediction.

| 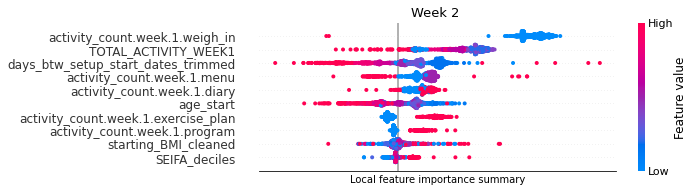  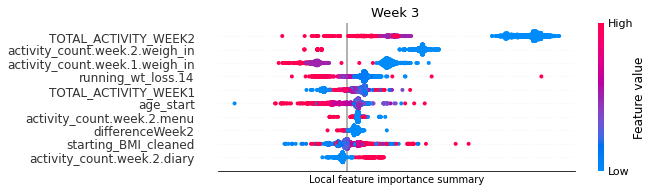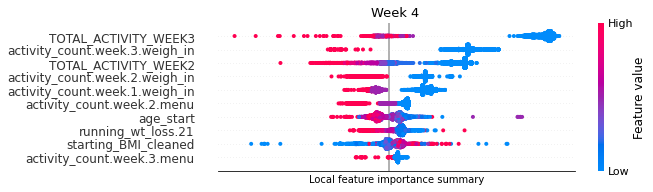  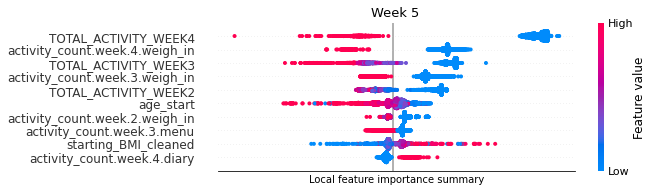  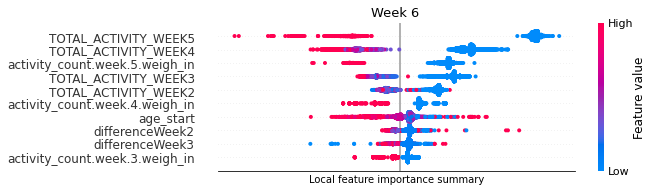  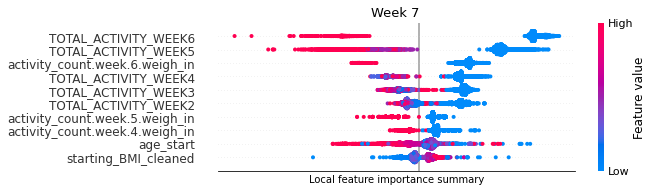  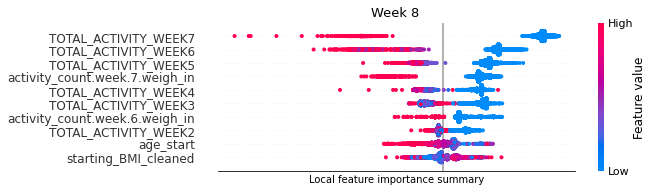  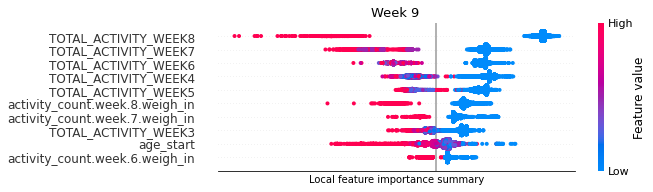  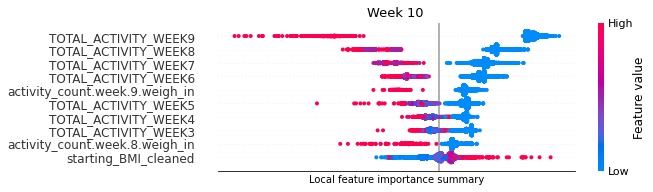 |
| --- |

| 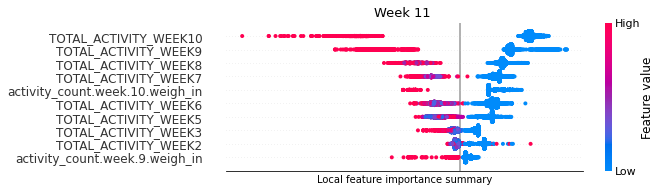  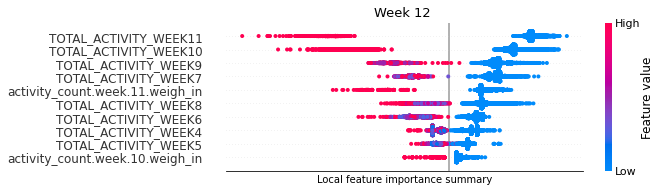 |
| --- |

Figure S8. Summary plot of Shapley values obtained on test data for each predictive model
